# Development of an Exercise Training Protocol to Investigate Arteriogenesis in a Murine Model of Peripheral Artery Disease

**DOI:** 10.3390/ijms20163956

**Published:** 2019-08-14

**Authors:** Ayko Bresler, Johanna Vogel, Daniel Niederer, Daphne Gray, Thomas Schmitz-Rixen, Kerstin Troidl

**Affiliations:** 1 Department of Vascular and Endovascular Surgery, University Hospital Frankfurt, Theodor-Stern-Kai 7, 60590 Frankfurt, Germany; 2 Department of Sports Medicine, Institute of Sport Sciences, Goethe University, Ginnheimer Landstraße 39, 60487 Frankfurt, Germany; 3 Department of Pharmacology, Max-Planck-Institute for Heart and Lung Research, Ludwigstr. 43, 61231 Bad Nauheim, Germany

**Keywords:** arteriogenesis, exercise training, mouse model, femoral artery ligation, running wheel, voluntary training, peripheral artery disease

## Abstract

Exercise is a treatment option in peripheral artery disease (PAD) patients to improve their clinical trajectory, at least in part induced by collateral growth. The ligation of the femoral artery (FAL) in mice is an established model to induce arteriogenesis. We intended to develop an animal model to stimulate collateral growth in mice through exercise. The training intensity assessment consisted of comparing two different training regimens in C57BL/6 mice, a treadmill implementing forced exercise and a free-to-access voluntary running wheel. The mice in the latter group covered a much greater distance than the former pre- and postoperatively. C57BL/6 mice and hypercholesterolemic ApoE-deficient (ApoE^−/−^) mice were subjected to FAL and had either access to a running wheel or were kept in motion-restricting cages (control) and hind limb perfusion was measured pre- and postoperatively at various times. Perfusion recovery in C57BL/6 mice was similar between the groups. In contrast, ApoE^−/−^ mice showed significant differences between training and control 7 d postoperatively with a significant increase in pericollateral macrophages while the collateral diameter did not differ between training and control groups 21 d after surgery. ApoE^−/−^ mice with running wheel training is a suitable model to simulate exercise induced collateral growth in PAD. This experimental set-up may provide a model for investigating molecular training effects.

## 1. Introduction

Patients with peripheral arterial disease (PAD) often suffer from intermittent claudication, leading to a significant walking impairment. According to the 2017 guidelines of the European Society of Vascular Surgery (ESVS), supervised exercise training is a Class I, Level A recommendation in patients presenting intermittent claudication, whereas unsupervised training is a Class I, Level C recommendation. Walking has thus been shown to be a safe and effective treatment for patients with PAD [1,2]. In particular, walking performance, cardiovascular parameters, and quality of life can be improved by exercise.

Some potential exercise effects and scheduling modifiers are still unclear, in particular, risk factors such as smoking, dyslipidemia, diabetes mellitus, obesity, and arterial hypertension as major comorbidities of PAD are only considered infrequently [3,4,5,6]. Physical activity has suppressive effects on inflammation [7] and proinflammatory immune cells [8,9], as well as beneficial effects on endothelial function [10]. Additionally, physical training has the potential to promote an additional vascularization in hypoxic/ischemic tissues, such as the myocardium or peripheral limb [11]. Arteriogenesis, can be induced by exercise in human [12,13,14] and in animal studies [15]. The driving force of arteriogenesis is altered fluid shear stress (FSS) in the preformed collateral arteries due to increased blood flow [16]. The increased blood flow initiates vascular remodeling and diameter growth [17] and alters the miRNA profile [18].

Nevertheless, the physiological pathways of how exercise affects collateral growth at the molecular level are still not finally delineated.

Arteriogenesis is the process that results in growth of pre-existing collateral arterioles into functional collateral arteries, triggered by a hemodynamically relevant stenosis of supplying blood vessels. These bypassing vessels can sometimes be remarkably efficient and nearly completely replace the occluded arteries [19]. This formation is stimulated by an increase of shear stress on the endothelium [20]. An increase of blood flow can be achieved by a high demand and walking exercise gives the best possibility to maximize the flow physiologically [6].

In past decades various models have been developed that help in understanding the mechanisms of arteriogenesis. The ligation of the femoral artery (FAL) in mammals, especially the mouse, has become a well-established model for the induction of arteriogenesis [3,21,22]. Exercise stress tests are widely used for a variety of training protocols [23,24,25]. In most of the mice models, the training is voluntary (treadmill or running wheel), only a minor share of the protocols is forced. As exercise characteristics like frequency, intensity, type, and time cannot be controlled, a forced protocol may be appropriate. On the other hand, forced exercise is, unlike voluntary exercise, affected by distress [25,26,27].

The aim of this study was to find a suitable mouse model for simulating PAD as well as to establish a training protocol that would be accepted by cardiovascular-diseased animals and stimulate arteriogenesis. Such a protocol could provide the basis to methodically investigate effects of training on PAD at the molecular level in an experimental setup.

## 2. Results

### 2.1. Evaluation of a Training Regime in C57BL/6

**Figure 1:**
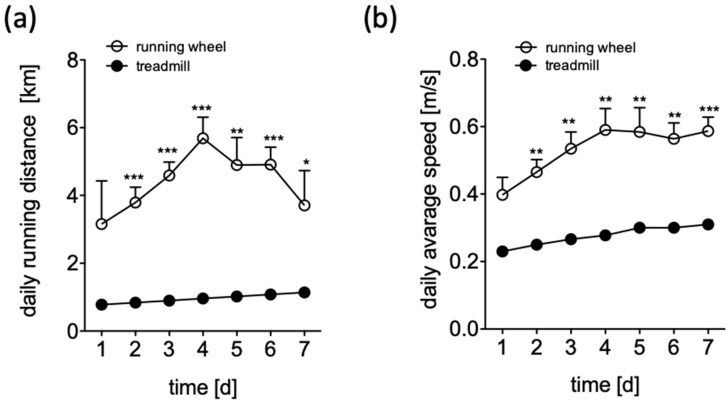
Daily running distance (**a**) and daily average speed (**b**) of C57BL/6 mice over time, during 7 days of adaptation in a treadmill receiving forced exercise (filled circles) or in a voluntary running wheel (open circles). Data are displayed as mean ± SEM. * *p* < 0.05; ** *p* < 0.01; *** *p* < 0.005.

In order to maximize training intensity, we compared two training regimens: A treadmill to implement forced exercise and a free-to-access voluntary running wheel. During the treadmill protocol the mice were trained using an incremental protocol: initial running speed was 0.2 m/s, increased daily to the maximal speed of 0.3 m/s at day 7. The training frequency was twice per day, and each training lasted 30 min. A maximum distance of 1.08 km was covered each day. Distances traveled and running wheel speeds were recorded continuously. After an adaptation period a running distance of 4.36 ± 0.41 km/d with an average speed of 0.5 m/s of was recorded. We observed increased speed and distance values for the voluntary exercise mice when compared to those forced to exercise in a treadmill throughout the observation period (Figure 1a,b).

### 2.2. Acceptance of Voluntary Training in ApoE^−/−^ Mice after HFD and Post-Surgery

**Figure 2:**
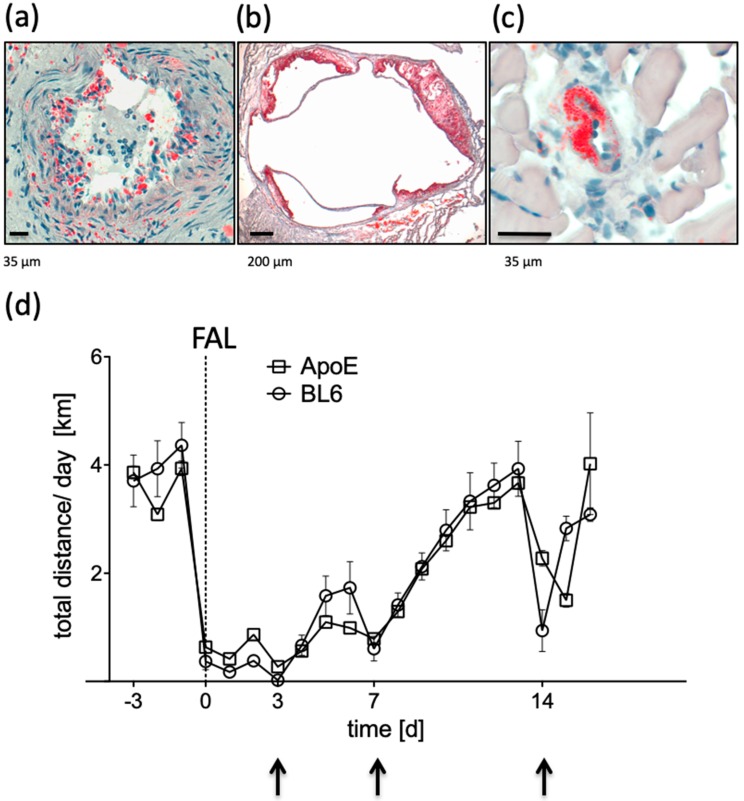
Plaque development in (**a**) the femoral artery, (**b**) the aortic root, and (**c**) the collateral artery of ApoE^−/−^ mice following 12 weeks of high fat diet, as visualized by Oil-red-O staining. (**d**) Daily running distances of C57BL/6 mice (BL6, circles) compared to ApoE^−/−^ mice (ApoE, squares) over time, pre- and post-ligation of the femoral artery (FAL). Arrows indicate short term running breaks due to anesthesia during perfusion measurements.

To further increase a similarity to patients presenting PAD we included ApoE^−/−^ mice fed with a high fat diet (HFD; 21% butter fat, 1.5% cholesterol) to the exercise study. These mice showed numerous plaques throughout the whole arterial system including the femoral artery and the aortic root as well as fat deposition in the collateral arterioles (Figure 2a–c).

Given the health constraints of ApoE^−/−^ mice following 12 weeks of HFD, we intended to evaluate whether they were able to cover the same distance as healthy C57BL/6 mice pre- and post-FAL. During the adaptation the mean distance covered was 3.93 ± 0.28 km/d which was not significantly different to C57BL/6 mice (*p* = 0.36). Post-FAL the re-adjustment period was similar to C57BL/6 mice, with no significant difference, as visualized in the equal progression of running performance (Figure 2d). Both strains, before and after surgery, recovered quickly to their initial distance when they had access to a voluntary running wheel.

### 2.3. Reperfusion Recovery after FAL in C57BL/6 and ApoE^−/−^ Mice with and without Training

**Figure 3:**
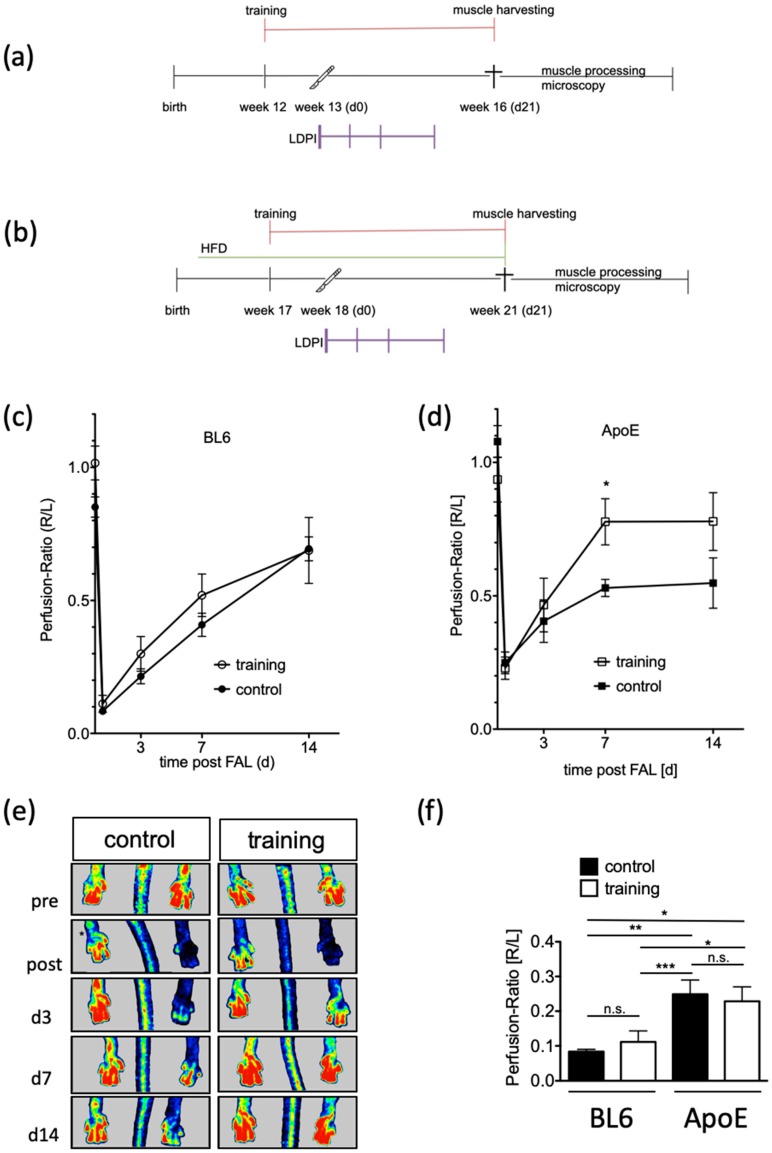
Functional effects on hind limb perfusion in response to training. (**a**) Schematic of experimental setup in C57BL/6, (**b**) schematic of experimental setup in ApoE^−/−^, and (**c**,**d**) laser Doppler perfusion imaging in C57BL/6 and ApoE^−/−^ as indicated. Data are expressed as ratio of the operated leg to the non-operated leg and represent mean ± SEM. Open symbols show the data of the training group whereas filled symbols represent the control group. As a statistical test, the unpaired t-test was used; * *p* < 0.05. (**e**) Representative laser Doppler perfusion images indicate the effect of training in the operated hind limb when compared to the control group. (**f**) Postoperative perfusion ratio (R/L). Open symbols show the data of the training group whereas filled symbols represent the control group. As a statistical test, the one-way ANOVA was used; * *p* < 0.05; ** *p* < 0.01; *** *p* < 0.005; n.s. not significant.

In order to evaluate training effects on reperfusion recovery, 18-week-old ApoE^−/−^ mice that had been fed with an HFD for 12 weeks and 12-week-old healthy C57BL/6 mice were subjected to the experimental protocols shown in Figure 3a,b. Both mouse strains were randomly subdivided into training or control groups. The control group was housed in motion-restricting cages. The training group was held in single cages containing a free-to-access running wheel starting 7 days prior surgery. FAL was performed on day 0 and perfusion of the hind-limb was measured by LDPI pre- and postoperatively and on postoperative days, d3, d7, and d14. Adductor muscle tissue was harvested from ApoE^−/−^ mice at different time points following FAL. The ratio of hind-limb perfusion in the operated leg to that in the non-operated leg dropped to 10% on average in C57BL/6 mice (training: 11 ± 4%, *n* = 12, control: mean 8 ± 1%, *n* = 16; *p* > 0.05), whereas perfusion recovery increased similarly within 14 days in the control (69 ± 10%) and training group (69 ± 4%) (Figure 3c).

In contrast, exercising ApoE^−/−^ mice showed a significantly faster approximation of perfusion with training (*n* = 19). A maximum of 78 ± 8% perfusion of the hind limbs could be reached within 7 days postoperatively, compared with the control group (*n* = 21) averaging 53 ± 3% (*p* = 0.012) (Figure 3d,e). Interestingly, immediately postoperatively ApoE^−/−^ mice showed a baseline perfusion of 25% independent of a training adaptation that was significantly higher than that of C57BL/6 mice (*p* = 0.001) (Figure 3f).

### 2.4. Increased Accumulation of Macrophages after Training in ApoE^−/−^ Mice

**Figure 4:**
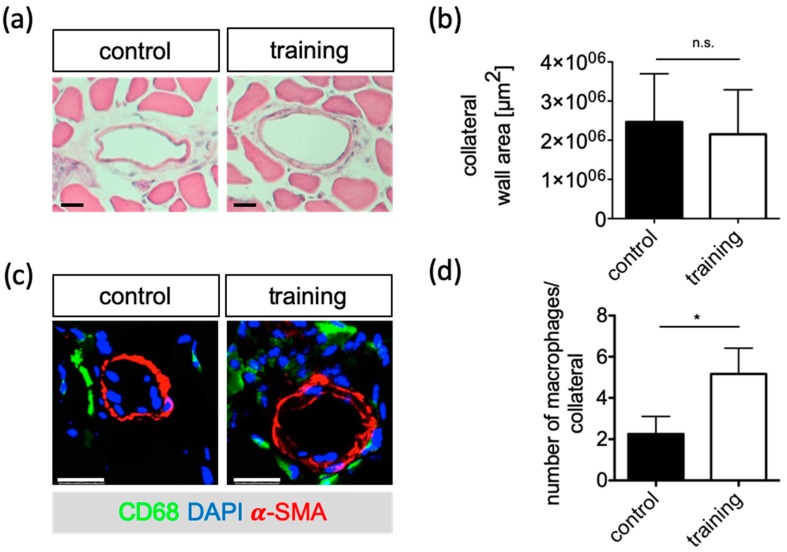
Histological evaluation of collateral growth in cross sections of the adductor muscles of ApoE^−/−^ mice in response to training. (**a**) Representative micrographs of collateral arteries for morphometry. Scale bar: 200µm. (**b**) Quantification of wall area 21 days after FAL of the training and control group. (**c**) Immunostaining to determine macrophage accumulation around collaterals 7 d after FAL of the training and control groups. Representative images of CD68 (green) and αSMA (red) immunostaining. Blue staining indicates nuclei and scale bars are 25 µm. (**d**) Quantification of macrophage number. Data are expressed as mean ± SEM of three collateral cross-sections per mouse of at least three mice per group (*n* ≥ 3). As a statistical test, the unpaired *t*-test was used; * *p* < 0.05.

In order to investigate the beneficial influence of exercise training on the vascular remodeling process in ApoE^−/−^ mice, adductor muscle tissue was harvested from these mice 21 days following FAL. Early perfusion benefits were not reflected by morphometric examination at the end of the experimental period. There was no difference of the size of the wall area between training and control groups on day 21 post-surgery (2.15 ± 0.53 mm^2^ and 2.46 ± 0.53 mm^2^, respectively; *p* > 0.05; Figure 4a,b).

In the initial phase, collateral growth is critically driven by pericollateral macrophage assembly. Therefore, adductor muscle tissue was harvested from ApoE^−/−^ mice 3 or 7 days following FAL and the macrophage number was quantified in the vascular nerve sheath of the collateral vessels. Seven days after FAL the number of macrophages in close proximity of growing collaterals was significantly higher in the training group with an average of 3.9 ± 0.8 compared to the control group with an average of 2.3 ± 0.4 (*p* = 0.042; Figure 4c,d).

## 3. Discussion

Arteriogenesis is the natural compensation mechanism through which collateral circulation develops. This formation is stimulated by an increase of shear stress on the endothelium [20]. An increase of blood flow can be achieved by a high demand and walking exercise is the best possibility to maximize the flow physiologically [6]. Therefore, the aim of the study was to establish a training protocol in a murine model of PAD that increases arteriogenesis through exercise. Our results suggest that a FAL surgery in ApoE^−/−^ mice having free access to a voluntary running wheel serves best for future studies of exercise-induced arteriogenesis.

To increase physiologic shear stress on the arterioles it is preferable to develop an exercise program that maximizes intensity.

Initially we compared activity of healthy young C57BL/6 mice trained either with forced exercise by treadmill twice daily or with a 24 h accessible running wheel. Forced exercise is controlled and reproducible, but usually depends on a negative impulse. In models with forced exercise regimens, increased distress values, depressive behavior, inflammation reactions, and elevated corticosterone levels have been shown [26,28,29,30]. Distress, for example, may limit physiological remodeling normally associated with exercise training in humans [31,32]. The transferability of forced exercise models into humans may thus be limited. In contrast, similar structural and functional cardiac changes occurred in forced and voluntary exercise regimens [33]. The pros and cons of a forced versus a voluntary exercise model are thus not finally delineated.

We further showed that voluntary training leads to a much greater distance covered than forced exercise. Furthermore, voluntary running resulted in a higher running speed. Resulting from this higher exercise dose, a higher training effect response of voluntary running than of forced treadmill walking would be expected. Free-to-access running wheels are an easy way to record and store activity data without disturbing the habitual behavior. Likewise, voluntary access to running wheels permits reasonable adaptation to exercise after surgery and provides an excellent tool to monitor the behavior of mice. We could show that mice do tolerate this voluntary training much better with an increase of the distance travelled compared to forced exercise. For the reasons given above we continued our studies with voluntary training knowing well that this cannot be directly translated to the human situation. There is a discrepancy in intrinsic exercise capacity and response to exercise training between mice and humans. Mice do have a natural drive for running. Humans with sedentary behavior do not push their maximum limit. The focus of our study was to establish a protocol in mice which was adapted to their natural behavior and allowed for future investigations on collateral growth.

Since in PAD a stenosis is progressing over time and involves the whole arterial system, there is an uncertainty if healthy animals can be used to simulate the human patient’s illness [3,23,34,35]. An acute occlusion in healthy participants by e.g., arterial emboli or trauma demands an instant intervention. The sudden tissue hypoxia can lead to an acute inflammatory–angiogenic–myogenic response which could result in massive loss of tissue. Patients suffering from PAD usually better tolerate an acute occlusion [36].

In order to increase the similarity to patients presenting PAD we used ApoE^−/−^ mice fed with a HFD, that show numerous plaques throughout the whole arterial system [21,23,24,37] including fat deposition in collaterals.

It was expected that different mouse strains don’t have similar responses to voluntary training [27,38]. Our findings showed that C57BL/6 and ApoE^−/−^ animals accepted the voluntary training without a notable difference between the two strains. In this study we showed that there was just a short postoperative readjustment period of 10 days needed to get mice back to the initial distance. Whether this delay was due to the surgical intervention alone (opening and closing of the skin) cannot be fully excluded, because a sham treatment group without FAL was not investigated.

Next, the voluntary training protocol (running wheel) was tested in both strains to evaluate the reperfusion recovery after FAL.

The LDPI data acquired showed a significant higher perfusion immediately after the occlusion in ApoE^−/−^ mice. This could be explained due to an increased collateral growth as a result of arteriosclerotic plaques in the major arteries [21]. C57BL/6 as well as ApoE^−/−^ mice presented a maximum re-perfusion up to 69% and 77% with no significant difference in between the two strains.

It could be shown that having training possibility allowed ApoE^−/−^ mice to reach the maximum reperfusion alignment one week post-FAL.

In order to correlate the increased perfusion to arteriogenesis we performed histological analyses of collateral tissue of ApoE^−/−^ mice with and without exercise training. It is well accepted that mechanical, cellular, and molecular factors influence collateral growth [39]. Macrophages accumulate around the growing collaterals and cytokine secretion improves that process [40,41]. After training, ApoE^−/−^ mice show a higher accumulation of CD68^+^ macrophages in the vascular nerve sheath of the collateral vessels than without training.

The presented experimental setup involves atherosclerotic ApoE^−/−^ mice subjected to an acute FAL. Functional as well as histological findings implicate an improvement of arteriogenesis after exercise training in the proposed model.

## 4. Materials and Methods

### 4.1. Ethics Statement

Animal handling and all experimental procedures carried out were in full compliance with the Directive 2010/63/EU of the European Parliament on protection of animals used for scientific purposes. Approval was given by the responsible local authority, the Darmstadt governmental council for animal protection and handling (permit reference numbers V54-19c20/15-B2/360, permit date: 30 October 2013). Throughout this study all mice had access to water and food ad libitum.

### 4.2. Femoral Artery Ligation (FAL)

Twenty-eight male C57BL/6 mice (Charles River, Sulzfeld, Germany) and 40 male ApoE^−/−^ mice were subjected to FAL as described [22]. The contralateral leg served as the reference. During the surgical procedure mice were kept on a heating plate with a temperature of 38 °C. Anesthesia was applied using ketamine (120 mg/kg BW) and xylazine (16 mg/kg BW) i.p.. For postoperative analgesia carprofen (5 mg/kg BW) was injected s.c.. After termination of experiments the mice were euthanized by an anesthetic overdose.

### 4.3. Forced Exercise on Treadmill

Mice were first accustomed by using a treadmill (Exer 3/6, Columbus Instruments, Columbus, OH, USA) with a motivation grid for 15 min/day. This applied small amounts of electric shock for conditioning when the mice stopped running.

After being conditioned, the mice started training at a light intensity with a preset speed of 0.2 m/s leading up to a maximum of 0.3 m/s with no further resistance. Training frequency was 2 times per day. Training was terminated at exhaustions. Exhaustion was defined as pause of more than 5 s at a time, or three times for two or more seconds on the shock pad without trying to get back on the treadmill.

### 4.4. Voluntary Running Wheels

To evaluate voluntary training each animal was individually housed in a cage equipped with a free-to-access running wheel. The running wheels were connected to a computer equipped with TSE PhenoMaster V5.1.6 (2014-4115) (TSE Systems GmbH, Bad Homburg, Germany). This setup gave accurate data on each animal, recording time and traveled distances and it allowed evaluation of activity patterns during an adaptation period as well as identifing post-surgery effects.

### 4.5. Restraining Cages

For simulating inactivity, mice were kept as a reference (control) group in smaller cages without the possibility to climb in order to minimize movement.

### 4.6. Laser Doppler Perfusion Imaging (LDPI)

Perfusion of the paws was recorded using the laser Doppler imaging device PIM3 (Perimed Instruments, Järfälla, Sweden; Software: LDPIwin for PIM3 3.1.3), on a heating plate at 38 °C, before FAL (d0 pre), immediately after FAL (d0 post), as well as at d3, d7, and d14 after FAL.

The mean perfusion was shown as the ratio of the ligated hind limb to the contralateral, non-ischemic hind limb.

### 4.7. Histology

Mice were perfused with 10 mL vasodilation buffer (100 µg adenosine, 1 µg sodium nitroprusside, 0.05% BSA in PBS, pH7.4) followed by 10 mL 4% PFA post mortem. Adductor muscles from ligated or sham-operated mice were harvested and placed in 15% sucrose in PBS for 4 h and overnight at 4 °C in 30% sucrose in PBS. Tissue was cryopreserved in Tissue-Tek O.C.T. (Sakura, Alphen aan den Rijn, The Netherlands) and cut in 8 µm cryosections. Morphometric analyses were performed using a hematoxylin-eosin stain to evaluate the dimensions of collateral arteries.

### 4.8. Immunohistochemistry

Cryosections were stained with blue-fluorescent DNA stain DAPI (4′,6-Diaminidino-2-phenyllindole-dilactate; Thermo Fisher Scientific, Waltham, MA, USA) and counterstained for αSMA-Cy3 (C6198, Sigma-Aldrich GmbH, Taufkirchen, Germany) or αSMA-FITC (F3777, Sigma-Aldrich GmbH, Taufkirchen, Germany) and CD68 (MCA1957A448T, AbD Serotec, BioRad, Feldkirchen, Germany).

### 4.9. Statistical Analysis

All statistical analyses were supported by GraphPad software PRISM5 for Mac (GraphPad Software, La Jolla, CA, USA), JMP for Mac (Version 9.0.12010 SAS Institute Inc., Heidelberg, Germany), Image J (National Institutes of Health, Bethesda, Maryland, USA) and Microsoft Excel for Mac (Version16.15, Microsoft, Redmond, Washington, USA). Comparisons between groups were based on unpaired student’s t-test. One-way or two-way ANOVA were used to determine differences between the means of three or more independent groups as indicated. Data were reported as standard error of mean (SEM). Data deviations were considered to be statistically significant differences at *p* < 0.05.

## 5. Conclusions

Femoral artery ligated ApoE^−/−^ mice on HFD with running wheel training is a suitable model to simulate exercise induced collateral growth. This experimental set-up may provide a model for investigating molecular training effects on arteriogenesis.

## Abbreviations

PAD	Peripheral artery disease
CVD	Cardiovascular disease
ApoE^−/−^	Apolipoprotein E knockout
FAL	Femoral artery ligation
HFD	High fat diet
